# Treatment-Free Remission in Chronic Myeloid Leukemia: Can We Identify Prognostic Factors?

**DOI:** 10.3390/cancers13164175

**Published:** 2021-08-19

**Authors:** Hilbeen Hisham Saifullah, Claire Marie Lucas

**Affiliations:** 1 Chester Medical School, University of Chester, Bache Hall, Chester CH2 1BR, UK; 2 Department of Molecular and Clinical Cancer Medicine, University of Liverpool, Liverpool L69 3GA, UK

**Keywords:** chronic myeloid leukemia, chronic phase, tyrosine kinase inhibitor, treatment-free remission, TFR deep molecular response, BCR–ABL

## Abstract

**Simple Summary:**

Chronic myeloid leukemia (CML) is a blood cancer. Unlike other cancers CML treatment is lifelong and many patients experience side effects. For those patients who respond well to treatment and achieve deep molecular remission, quality of life is impacted because of continuous treatment. In this review, we look at emerging clinical trials which aim to investigate which patients can safely stop treatment. Treatment-free remission is the ultimate goal for CML patients, but there is still a gap in our knowledge as to why some patients can achieve treatment-free remission, while others relapse when treatment is stopped. Here we discuss if there are any prognostic factors that can predict the best candidates who qualify for treatment discontinuation, with a view to keeping them in remission.

**Abstract:**

Following the development of tyrosine kinase inhibitors (TKI), the survival of patients with chronic myeloid leukaemia (CML) drastically improved. With the introduction of these agents, CML is now considered a chronic disease for some patients. Taking into consideration the side effects, toxicity, and high cost, discontinuing TKI became a goal for patients with chronic phase CML. Patients who achieved deep molecular response (DMR) and discontinued TKI, remained in treatment-free remission (TFR). Currently, the data from the published literature demonstrate that 40–60% of patients achieve TFR, with relapses occurring within the first six months. In addition, almost all patients who relapsed regained a molecular response upon retreatment, indicating TKI discontinuation is safe. However, there is still a gap in understanding the mechanisms behind TFR, and whether there are prognostic factors that can predict the best candidates who qualify for TKI discontinuation with a view to keeping them in TFR. Furthermore, the information about a second TFR attempt and the role of gradual de-escalation of TKI before complete cessation is limited. This review highlights the factors predicting success or failure of TFR. In addition, it examines the feasibility of a second TFR attempt after the failure of the first one, and the current guidelines concerning TFR in clinical practice.

## 1. Introduction

Chronic myeloid leukaemia (CML) is a heterogeneous haematopoietic disorder and is considered the first model in cancer for targeted therapy [1]. Discovery of the Philadelphia chromosome [2,3] and the *BCR*–*ABL1* gene [4] and the development of tyrosine kinase inhibitors made a quantum leap in the treatment of CML. The natural history of CML makes it a unique disease that arises from leukaemic cells, and then progresses from chronic status through an accelerated phase to blast crisis [5]. Moreover, it was reported that CML exhibits the oncogenic addiction phenomenon, that is BCR–ABL through its kinase activity is the weakest point of the leukaemic cells [6]. However, recent research has illustrated that leukaemic stem cells (LSC) do not depend on the *BCR*–*ABL1* gene [7].

*BCR–ABL*1 became a therapeutic target for leukaemic cells and paved the way for the development of tyrosine kinase inhibitors (TKI) that dramatically improved the survival of CML patients [8,9]. Furthermore, patients with CML were achieving life expectancy near to that of the non-CML population [10,11,12]. However, now the expectations are rising for patients with CML switching from achieving long-term survival to stopping the treatment [13]. In the past, it was reported that a small number of patients on Interferon-Alpha could stop the treatment; nowadays, the same concept is being suggested for the patients taking TKI [14].

In most cases, patients on TKI, achieve deep molecular response (DMR) which improves the survival rates and reduces the chance of disease progression in the future [15,16,17]. The ELN defines DMR as undetectable *BCR*–*ABL1* mRNA transcripts in the blood using quantitative reverse transcription polymerase chain reaction (qRT-PCR) and/or two consecutive high-quality PCR samples with >10^4^ sensitivity [18]. According to international scale, deep levels of molecular response is defined as MR^4^ indicates >4.0-log reduction (*BCR*–*ABL1*^IS^ < 0.01%), MR^4.5^ indicating >4.5-log reduction (*BCR*–*ABL1*^IS^ < 0.0032%), and MR^5^ which indicates >5.0-log reduction (*BCR*–*ABL1*^IS^ < 0.001%) [19]. Discontinuing TKI, after achieving a DMR will be a novel approach to optimise the treatment and minimising the burden on healthcare systems and patients [20]. This review aims to discuss the concept, mechanism, and trials of treatment-free remission (TFR) and reviews potential predictors of the success or the failure of TFR. Furthermore, it aims to show how far the concept of TFR can be clinically applicable.

## 2. The Concept of TFR

**Figure 1:**
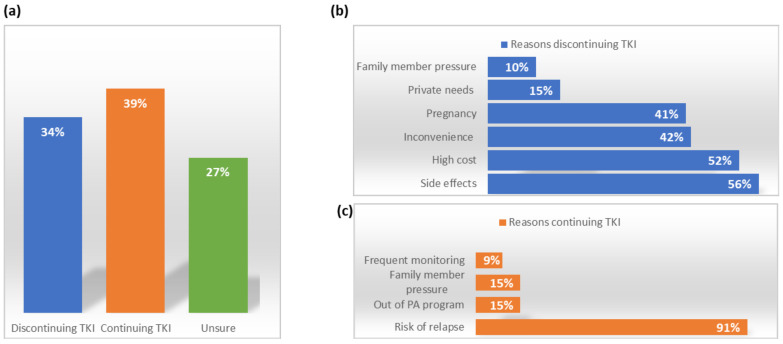
Percentage of patients willing to attempt TFR and the reasons given: (**a**) Percentage of patients willing to attempt TFR; (**b**) The reasons for discontinuing—the main reason that patents cited for wanting to for stopping TKI is the side effects; and (**c**) The reasons for continuing TKI treatment—the main reason patients wanted to continue treatment was fear of relapse. Adapted from [27].

Professor John Goldman introduced the concept of the operational cure for patients with CML years ago to indicate that patients can have a controlled disease, and a normal to near-normal life while continuously taking the TKI [21]. However, TFR takes this concept further by proposing that patients can have a healthy life and controlled disease without the need to continue the treatment after achieving DMR [22]. Although TKI are well-tolerated and have mild to moderate toxicity, a significant proportion of patients have side effects that interfere with their quality of life [23]. Furthermore, newer generations of TKI are more powerful and associated with severe adverse events, such as vascular events, myocardial infarction, pleural effusion, and pulmonary hypertension [14]. Around 30% of patients experience side effects from TKI that interfere with their daily activities and which on a long-term basis can lead to a decrement in quality of life [24,25]. This is quite true for younger patients aged 18–39 as TKI impact their lives more than that of older patients [26]. In a multi-centre survey on 329 CML patients, 34% of the responders were willing to discontinue TKI, and the main reason was the low quality of life, while 31% of the participants did not want to stop the TKI because of relapse risk [27] (Figure 1). Moreover, the survey showed that younger patients, are most likely to attempt TFR [27]. Moreover, pregnant women cannot take TKI as this class of medications is contra-indicated during pregnancy. Although CML is very rare in children, continuously taking TKI could affect their growth [1]. Nonetheless, maintaining a good quality of life and therapy cost represent the main two issues of CML patients, and the question to discontinue therapy is raised [28,29].

## 3. Clinical Trials of TFR

**Figure 2:**
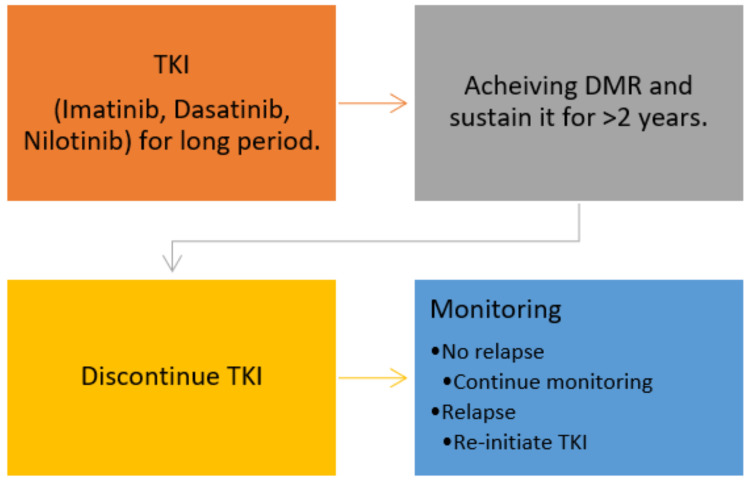
TFR concept, patients achieving DMR after long-duration on TKI can attempt for TFR, with continuous monitoring, those who relapse will re-initiate the treatment. Abbreviations: TKI = tyrosine kinase inhibitor, DMR = deep molecular response, and TFR = treatment-free remission.

The first stopping pilot study was conducted in 2004 in France by Professor Mahon and colleagues [30]. Clinical trials of TFR are limited with the concept that patients on long-term TKI who achieved a durable molecular response can discontinue TKI (Figure 2). However, most TFR trials have limitations. Firstly, all current trials are non-randomised trials except for the HOVON trial, in which patients were randomised into two arms after achieving MR^4.5^ for >2 years. Arm A includes patients who continue Imatinib, and arm B patients discontinuing Imatinib [31]. Molecular relapse in arm A was 17% while in arm B 67%. Moreover, most of TFR trials are observational studies and in these types of studies the risk of selection bias is increased [32]. A survey reported that 56% of patients had anxiety and fear of discontinuing TKI [33]. This anxiety and fear affect the selection criteria and participation of patients in TFR trials.

Furthermore, the small population size of the trials is another limitation except for the EURO-SKI, which is the largest trial for TFR to date [34]. The EURO-SKI is conducted in 11 countries and 61 European centres, including 755 CML patients. Small study samples yield false-positive results, and there is a challenge with data interpretation. The absence of a generalised and comprehensive definition of undetectable minimal residual disease (UMRD) and complete molecular response (CMR) made the comparison between the trials difficult as it depends on the sensitivity qRT-PCR which is not consistent across trials. Moreover, eligibility criteria to initiate TFR is different from one study to another. For example, molecular response which is a crucial aspect for TFR feasibility, the definition and level of response, and trigger for re-initiation of TKI are not standard between the trials (Table 1).

Table 1 demonstrates the characteristics of the trials initiating TFR. These presented trials comprise a proportion of data related to TFR that can be of significance in the future. What can be observed from Table 1, is the overall TFR rate can be estimated between 40–70%. Almost all trials require a minimum of two years of treatment with TKI. Moreover, the duration of DMR needs to be at least two years. Furthermore, most of the molecular relapses occur early within the first six months after TKI discontinuation, potentially suggesting that residual clones are molecularly similar to the original disease.

## 4. Mechanisms behind TFR

To date, mechanisms underlying success or failure of TFR are unknown but research suggests that the biology of leukaemic stem cells (LSCs), the microenvironment, and the role of the immune system are key [20]. In vitro experiments reported that LSCs are resistant to TKI therapy, and haematopoietic stem cells (CD34+/CD38-cells) do not solely depend on *BCR*–*ABL1* for survival [7,56]. With these results, it is important to ask whether long-term TKI therapy will eliminate LSCs and is it possible to impose the elimination of LSCs as a requirement for TKI cessation? Chomel et al. reported that LSCs are persistent in CML patients with UMRD, treated with prolonged IM therapy demonstrated by Ph+ culture initiating LSCs [57]. Moreover, the same results were confirmed by Chu et al. through *BCR*–*ABL1* expression in CD34+/CD38-cells [58]. These results suggest that LSCs remain after long-term TKI therapy, and dynamic models of CML suggest that may would take >20 years of IM treatment to eradicate LSCs but this has not been proven [59].

Another mechanism explains the role of the immune system in maintaining DMR and successful TFR. Before the introduction of TKI, Interferon (IFN) therapy-induced immune activation and targeting stem cells by cytotoxic stem cell survival, enhanced natural killer (NK) cells cytotoxicity [60,61]. Three studies further confirmed the role of the immune system in controlling CML after TKI discontinuation by demonstrating low NK cells as predictors for molecular relapse [62,63,64]. On the other hand, those with defective immune systems have an increased risk of virus-related carcinomas and other malignancies but not CML [65]. Whether this mechanism occurs in specific cases or in relation to other factors, the fundamental role of the immune system needs further evaluation.

Another mechanism behind TFR concept is the latency that explains the interval in which BCR–ABL formation until the diagnosis of CML. This interval is heterogeneous and ranges between 2–40 years, following the atomic bombs at Hiroshima and Nagasaki, the incidence of leukaemia increased within 2–3 years [66,67]. Alternatively, this could be due to stochastic events in which stem cells simply do not divide; however, data showed that this mechanism could occur by chance [68]. Despite these hypotheses, it is essential to know that patients with persistent LSCs do not necessarily end with relapses and discovery of biomarkers would allow treatment to be safely discontinued.

## 5. Predicting Factors for the Success or the Failure of TFR

### 5.1. Duration of TKI

One of the most consistent factors across all trials that affect TFR is the duration of TKI. Most of the trials required a minimum of three years TKI to introduce a successful TFR. This factor seems consistent, and the longer the duration, the more chance of TFR success. In the STIM trial, the cut-off of Imatinib therapy was 50 months [35]. The estimated survival at 18 months without relapse was 22% for <50 months and 47% for >50 months TKI. Moreover, the median survival time without relapse was 2.8 and 5.5 months for both <50 months and >50 months TKI *p* = 0.033. A multivariate analysis of the factors predicting relapse duration of TKI considered a prognostic factor (hazard ratio 0.421 *p* = 0.010) [35]. After long-term follow-up, in the STIM trial, the duration of Imatinib remained an independent prognostic factor *p* = 0.024 [36]. For the EURO-SKI trial, the cut-off level of Imatinib was 5.8 years. TFR rate was 41% and 63% for <5.8 years and >5.8 years respectively with an odds ratio of 1.16, *p*-value = 0.0001 [34]. That means an increase by 16% of the MMR rate when an additional year of Imatinib is added [34].

Furthermore, the results from the TRAD trial were consistent with the previous results where data cut-off was 8.7 years [49]. The TFR rate for >8.7 years was 79.5% and 27.9% for those <8.7 years hazard ratio 0.198 *p*-value < 0.001 [49]. Furthermore, Fava et al. reported that for each additional year of Dasatinib, there is a 22% decrease in relapse risk [45]. Moreover, KID, DOMEST, and DASFREE studies reported that longer TKI duration was associated with better TFR rate [40,53,55].

### 5.2. Deep Molecular Response

The depth or duration of molecular response may be an indicator for the success or failure of TFR. Sustained UMRD is defined as the absence of *BCR*–*ABL1* transcript from bone marrow or blood sample [57]. In single case studies, it was reported that sustained molecular response is related to more disease relapses after TKI cessation [69,70,71]. Furthermore, many studies reported that the depth of DMR and sustainable UMRD are related to the success rates of TFR [30,44,46,47]. However, a trial named ‘According to Stop Imatinib’ (A-STIM) less strict eligibility criteria were used as patients with occasional low levels of *BCR*–*ABL1* transcripts were also included [38]. Furthermore, the re-initiation of TKI criteria was the only loss of major molecular response (MMR < 0.1% *BCR*–*ABL1* transcript). With a median follow-up time of 31 months, cumulative molecular relapse was 35% and 36% at 12 and 24 months, respectively [38]. When using the STIM criteria, the relapse rate was 54%. Moreover, the relapse-free survival rate was 65% for those with stable UMRD and 64% for those with positive low levels of *BCR*–*ABL1* transcript prior to IM discontinuation [38]. If there is a deep response, this possibly means that there are fewer LSC, and so does a small pool of LSC favour TFR?

With these findings two points can be derived from this study: (1) low levels of the residual disease do not necessarily mean relapse and do not hinder the possibility of successful TFR, and (2) introducing TKI discontinuation for a larger group of CML patients is possible. The EURO-SKI trial also indicated no differences between the levels of molecular response and their relation to the TFR success rate. However, the long duration of DMR seems like a factor to predict the rate of TFR, although this has not been formally tested [37,40,50]. In the EURO-SKI trial, longer duration of DMR before TKI discontinuation related to a better chance of TFR [34]. The probability of staying in MMR at six months increased by 3% with each year of DMR, odd ratio (1.13 (1.04–1.23) *p* = 0.0032). The longer the duration of DMR, the better the chance of not having a relapse and the possibility of remaining in MMR at six months is 13% [34]. Takahashi et al. reported that there is a significant difference between the TFR rates of those with <24 months sustained DMR and those with >24 months DMR [72]. Nearly 78% for >24 months and 15% for <24 months *p* = 0.0002. However, with the multivariate analysis the duration of DMR did not occur as an independent prognostic factor [72].

Furthermore, in the TRAD trial cox progression model revealed that there is a strong relationship between long duration of MR^4^ or MR^4.5^ and relapse-free survival [49]. Nevertheless, the duration of DMR is related to the duration of TKI therapy that means both are confounding factors and increase over time (the longer duration of TKI, the longer DMR duration). This is why the levels of DMR and early molecular response are related to the sensitivity of TKI, and these results were further confirmed by the TWISTER study. [37] Around 68% of the relapsed cases occur in the first six months, and TFR rate at two years was 47.1% (95% CI, 31.5–62.7%). [37] Moreover, Fava et al. reported that patients in their observational study were characterised by the early response within three months, explaining the comparability of TFR rates between first and second line TKI [45].

### 5.3. BCRABL1 Transcript Type and Detection

Chronic myeloid leukaemia is driven by the *BCR*–*ABL1* gene which encodes for a p210 tyrosine kinase. It is known that there is variability in breakpoint position, the breakpoints of chromosome 22 cluster within a small (5.8 kb) region, spanning exons e12–16 known as the Major Breakpoint Cluster Region (M-BCR). Breakpoint locations almost always fall between either exons e13 and e14 or between e14 and e15. Although the breakpoints in the ABL gene are also variable, because of splicing events, the transcribed mRNA has either an e13a2 or an e14a2 junction. The e13a2 and e14a2 *BCR*–*ABL1* transcript types differ in length by 75 bp (25 amino acids) [73]. Previous reports prior to the introduction of imatinib have, in general, not identified an effect of *BCR*–*ABL1* transcript type on clinical outcome [74,75,76,77,78,79,80]. We have previously shown that imatinib-treated patients expressing the e14a2 transcript type have a higher and more rapid complete cytogenetic response rate than e13a2 expressing patients, thought to be due to higher BCR–ABL tyrosine kinase activity [81]. The rate of DMR been found to be lower in e13a2 patients [82,83,84,85,86]. In TFR studies, e13a2 are more likely to have molecular relapse than e14a2 patients. In the UK Destiny study, 15% of e13a2 patient relapsed compared to 7% of patients with e14a2 [87]. In a retrospective analysis of an Adelaide cohort, patients with e13a2 *BCR*–*ABL1* transcript had higher rates of molecular recurrence after TFR attempts, 66% vs. 35% [88]. These data suggest that e13a2 patients may need to be monitored more closely when attempting TFR.

Droplet digital PCR (ddPCR) is a more sensitive method for quantifying *BCR*–*ABL1* transcript levels. The *BCR*–*ABL1* levels assessed by ddPCR has been found to predict molecular recurrence when treatment is discontinued [89]. This supports the theory that the lower the *BCR*–*ABL1* level is the higher the chances or achieving a durable TFR, this was also observed in the UK Destiny study, which is the only study to include patients in MR3 and MR4 at trial entry [87].

### 5.4. Immunity Effect

This topic is controversial in relation to predicting the success or failure of TFR. It is suggested that the immune system in CML is not totally compromised and TKI helps re-activation of the immune system [90,91]. Several sub-studies in the first discontinuation studies discuss the relation of baseline immune markers as predictive factors for TFR. In preliminary results of Immunostim from the STIM revealed that the count of natural killer (NK) cells is lower in patients with relapse than those who maintained relapse-free survival (145/mm^3^ (67–450) versus 233/mm^3^ (70–727), *p* = 0.0146) [92]. However, NK receptor expression did not differ between the two groups [92]. This indicates a positive relationship between the NK cells in the peripheral blood and the rate of TFR, contributing to CML control. A sub-study to the EURO-SKI trial held by the Nordic CML study group (NCMLSG) reported that patients with high rates of mature (CD57^+^) and cytotoxic (CD16^+^ and CD57^+^) NK cells at the time of TKI discontinuation have higher possibility of successful TFR [93] and this suggests the importance of the surveillance of baseline immune markers before TKI discontinuation. It is thought that higher levels of NK cells may be capable of both directly killing the LSC and potentiating adaptive immune responses, thus maintaining remission after treatment discontinuation.

In the D-STOP trial, Dasatinib consolidation for two years revealed an increased rate of NK cells (CD3^−^CD56^+^) in patients who relapsed before Dasatinib cessation [52]. On the other hand, in the DADI (2015) trial, after one year of consolidation of Dasatinib, patients with increased counts of NK cells (CD3^−^CD56^+^ and CD16^+^/CD56^+^) and NK cell large granular lymphocyte (CD56^+^CD57^+^) numbers associated with a higher possibility of achieving successful TFR [41]. Furthermore, the DADI trial (2020) reported that low CD4 cell count prior to Dasatinib cessation is associated with successful TFR [42]. These different results can be due to different immune subsets that are introduced by different TKI regimen. For instance, in the DADI trial Dasatinib consolidation was used for one year, however in EURO-SKI and Immunostim, Imatinib was used. One of the possibilities discussed is the presence of Regulatory T (TReg) cells that help the tumour cells to invade the immune system and induces relapse in these group of patients. However, studies did not report any significant difference in Treg cell counts between patients relapsed and those who did not [92,94]. Yoshida et al. reported that Dasatinib inhibits Treg cells, and this is crucial for NK differentiation and to achieve DMR [95]. The normalisation of immune defects in patients with CML is believed to be due to the elimination of leukaemic cells when achieving DMR, rather than immune recovery affecting the molecular response [96].

### 5.5. TKI Resistance

In the STOP 2G-TKI study, 65% of the patients had a previous suboptimal response to Imatinib and 21.7% had resistance. The cumulative risk of relapse at 48 months for those with prior intolerance of resistance was 76.92% (CI95%, 57.11–100, *p* = 0.0023) and TFR rate was 23.08% [51]. In the DADI (2015) trial patients who switched to Dasatinib due to Imatinib resistance had only 7.7% TFR rate at 12 months [41], suggesting that patients with previous TKI resistance had higher rates of TFR failure and should not be included in TKI discontinuation trials.

### 5.6. Prognostic Scoring—Sokal, ELTS, and Eutos Score

The Sokal score is the first risk-adjusted score that classified CML patients into low-, intermediate-, and high-risk patients at diagnosis before initiating the treatment [97]. The score measures the percentage of blast cells, platelets, spleen size, and the patient’s age [98]. Studies reported that patients with a low-risk Sokal score have better progression-free survival (PFS) and overall survival (OS) [99,100]. In the STIM trial patients with low Sokal score had a better probability of maintaining a DMR at 12 months than those with high risk (*p* = 0.008) and reported the Sokal score as a prognostic factor for molecular relapse (hazard ratio 2.012, (1.252–3.234) *p* = 0.004) [35]. In the longer follow-up of STIM trial, the Sokal score remained an independent prognostic factor *p* = 0.024 [36]. Similar results were reported in the TWISTER study [37] and in the Korean survey of 14 CML patients [44]. However, in the TWISTER study, most patients with a low Sokal score were in the Interferon–Imatinib cohort, which could be a confounding factor. As a low Sokal score is associated with high TFR rate, multivariate analyses of factors affecting TFR showed the Sokal score an independent prognostic factor [45,55]. New prognostic scoring systems such as the European Treatment and Outcome Study for CML (EUTOS) may be a better scoring system to classify CML patients [101,102]. The EUTOS scoring system is established by the ELN, by analysing data from 2060 CML patients, who were treated using imatinib. Patients were divided into high risk and low risk groups stratified by their ability to achieve complete cytogenic response (CCyR) in 18 months and PFS [103]. More recently, the EUTOS long-term survival score (ELTS) has been developed to look at the risk of disease progression to the advanced phase [104]. In terms of TFR, ELTS score had a weak trend in predicting successful TFR [89].

### 5.7. Age

Most TFR trials reported no relationship between age at diagnosis and risk of relapse. However, an Italian observational study of TKI discontinuation reported age as a statistically significant variable that affects the rate of TFR. Younger patients are at higher risk of relapse than older patients (HR = 0.84 [95%CI 0.73–0.97] *p* = 0.02) [45]. The same results were confirmed by ISAV study with approximately the same age at diagnosis (49 years), patients <45 years are more susceptible to experience a relapse than those >45 years *p* ≤ 0.0001 [105]. However, in the DASFREE trial age >65 might help to predict which patients are more likely to remain in TFR [39]. Younger patients of CML usually have more aggressive disease, with high blast cell count and splenomegaly [106]. Moreover, younger patients have lower cytogenetic and molecular response rates in comparison to older patients [106]. Understanding these differences and the causes behind them would be helpful to enhance the outcomes further. These results suggest that older patients are more favourable to achieve successful TFR. These differences could be attributed to the differences in disease biology, more specifically LSC biology at different ages. HSC lose function with age [107,108]. Could this be that aged LSC are also less functional and less likely to drive relapse?

### 5.8. Prior Interferon-Alpha (IFN-a) Therapy

Only a few trials concluded the significance of the prior IFN-a therapy in relation to high TFR rate. In Japan, a retrospective study revealed that patients with previous IFN treatment are less likely to relapse within 12 months of TKI discontinuation, with the rate of TFR being 76% in patients with prior IFN therapy in comparison to only 23% in those with no IFN treatment [72]. Furthermore, IFN therapy was an independent predictor for molecular relapse within 12 months (OR 0.0419 (95%CI 0.0044–0.4035) *p* = 0.0060) [72]. Moreover, in the TWISTER study, patients in the IFN-Imatinib cohort had a better TFR rate 52% vs. 34% for those only in Imatinib cohort, and longer IFN duration associated with higher TFR rates *p* = 0.05. [37] In the A-STIM study, there was a lower rate of MMR loss in patients with previous IFN therapy. However, this trend was not significant and could not be analysed as an independent prognostic factor [38].

The increased rates of TFR in previously treated patients with IFN could be attributed to the selection bias (selecting patients with longer duration of treatment) and low biological risk in this cohort of patients [109]. Although the exact mechanism of action of IFN is not precisely clear, one of the proposed ways is the effect of IFN on the activation of immune effector cells and haematopoietic stem cells [110,111]. This opens up again the role of the immune system in maintaining TFR and the importance of immunosurveillance. Although a different entity, it is worth of mentioning that Burchert et al. reported that maintenance with IFN after TKI cessation improved the molecular response attributing this improvement to the activation of a proteinase-3-specific cytotoxic T Lymphocytes [112].

### 5.9. Re-Initiating TKI and Second Attempt TFR

Molecular relapse was the criteria to re-initiate TKI. However, the measurement of these criteria differs among the trials of TKI discontinuation. In STIM molecular relapse was defined as positive *BCR*–*ABL1* transcript in qRT-PCR at two successive assessments with a ratio of *BCR*–*ABL1* to ABL 10^−5^ or more [35]. The TWISTER study had a different definition, which is a single loss of MMR (>0.1% *BCR*–*ABL1*^IS^) or two consecutive samples at any value [37]. However, the A-STIM study changed the concept and defined molecular relapse as loss of MMR at any time which is a less stringent criteria [38]. Loss of MMR then was used by the EURO-SKI and other studies as a trigger for starting TKI again.

As demonstrated in almost all studies discontinuing TKI, patients who relapsed were still sensitive to TKI treatment. Although molecular triggers for re-initiating treatment differ among the studies, the vast majority of patients regained molecular response within six months. It is thought that relapse has the same disease kinetics as the original disease, this is because the LSC population is preserved and drives the disease upon relapse. The only case that progressed to a blast crisis after re-initiating TKI and gaining MMR for the second time was reported by the A-STIM study [38]. Thus, in this case, progression cannot be attributed to TKI cessation or related to TFR period, and these cases are called ‘sudden blast crisis’ [113,114]. Moreover, another case of nilotinib-resistant F359V *BCR*–*ABL1* kinase domain mutation was detected with loss of MMR in the ENESTfreedom trial. However, it could not be explained whether the case pre-existed TKI cessation or emerged during the TFR period [43].

The appropriate time to re-introduce TKI after molecular relapse is another critical issue that needs further follow-up. As recommended by the ELN group, re-initiation of TKI should be within less than one month of molecular relapse and followed-up every three months after regaining MMR and DMR [115]. Women who are of childbearing age should be tested for pregnancy before re-starting the TKI [116]. In cases of patients not responding to the re-introduction of TKI, the regimen should be changed to a more potent one.

Few data are published on a second attempt to discontinue TKI (TFR2) after relapse from the first attempt. In the RE-STIM trial, 70 patients attempted a second TFR after relapsing from the first one. All patients in this trial achieved MR^4.5^ for >2 years. TFR2 rates were 48%, 42%, and 35% at 12, 24, and 36 months, respectively. Moreover, no cases progressed to accelerated phase or blast crisis. The only predictor for a TFR2 was the time to molecular relapse in the first TKI discontinuation. Patients who remained DMR within the first three months of the first TKI discontinuation had a rate of TFR2 of 72% at 24 months, while those who relapsed in <3 months the rate of TFR2 was 36% [117].

The TRAD study also assessed the feasibility of TFR2 in patients who failed the first one. Results show that after treatment with Dasatinib and achieving MR^4.5^ for more than one year, the TFR2 rate was 21.5 ± 8.5% at six months [118]. Again, time to relapse of the first attempt was a strong determinant of TFR2 success. The rate of TFR2 at six months was 30% in patients relapsing within 3–6 months in the first attempt in comparison to only 9% for those who relapsed in <3 months [118].

Moreover, the TRAD study reported that patients who lost MMR at the first attempt had a faster molecular relapse after Dasatinib cessation compared to patients who lost MR4 [118]. In addition, patients with 5.5 log reduction or deeper in *BCR*–*ABL1* transcript had better TFR2 rate at six months (28.7%) versus 0% in patients with 4.5–5.4 log reduction *p* = 0.017 [118]. According to these results, a second TFR is feasible in patients who relapsed after the first one, and the duration of the first TFR is a very significant factor. Nevertheless, the above data suggests that to initiate TFR2, strict criteria should be applied.

### 5.10. Abrupt Discontinuation versus Gradual Discontinuation

It is reported that reducing the dosage of TKI in patients with deep molecular remission is safe [119], and it will not affect the long-term efficacy of TKI [120]. However, most of the TFR studies attempt to abrupt discontinuation of TKI. The British study of the De-Escalation and Stopping Treatment with Imatinib, Nilotinib, or Sprycel (Destiny) carried out a gradual decrease of the TKI to half of the dose for 12 months before discontinuing for 24 months. Patients with deep molecular response MR^4^ and patients with stable MMR were included in this study [121].

Results of this trial are quite exciting, at 36 months recurrence-free survival was 72% for MR^4^ group and 36% for MMR group [121]. Patients reported better overall symptoms and no cases of disease progression were recorded. The results of the Destiny trial are significant as the rate of TFR at three years is very high reaching up to 70% (all other trials reported TFR rate between 40–60%) and including patients with durable MMR is also an essential step as these group of patients are scarcely included in trials. However, the mechanism behind the benefit of de-escalation is still not precise, and more investigations are needed for patients with MMR achieving TFR.

## 6. Guidelines and Clinical Practice

There is a difference among published TFR studies in terms of inclusion criteria stopping and re-initiation of the treatment. Nonetheless, a few recommendations have been put together by experts, the ESMO guidelines, the NCCN, and the French chronic myeloid leukaemia study group (FCMLSG). Table 2 demonstrates some of the guidelines. Such practice should only be carried out in highly specialised centres where standardised molecular testing is conducted as recommended by ESMO [122]. Moreover, the NCCN group recommended that ceasing TKI outside clinical trials should only be performed if all criteria are met [123]. These guidelines have been recently reviewed by Professor Clark, a leading expert on treatment discontinuation [124].

It is important to know that when considering any patient for TFR attempt, they should be well-educated about the procedure, motivated, fully committed, and not pressured to stop TKI. It was reported in a survey that approximately 10% of patients discontinue TKI due to family pressure [27]. Moreover, the patient should be in the chronic phase taking TKI for an extended period, without any recorded resistance or suboptimal response. DMR should be achieved for at least two years, with no disease progression to blast crisis [14,102,103].

Furthermore, physicians should inform the patients about the effects of withdrawing TKI an entity known as TKI withdrawal syndrome. This syndrome usually appears as musculoskeletal pain, and studies reported that <30% of patients discontinuing TKI experience pain that may remain for months after withdrawing TKI [126,127]. In the KID study, TKI withdrawal syndrome has been related to a better chance to achieve TFR [40]. This can cause anxiety; patients should be reassured that this can be treated with either paracetamol or non-steroidal anti-inflammatory medications.

One of the significant obstacles that prevent the establishment of TFR outside the clinical trials is the need for frequent monitoring. In the first few months of stopping TKI, patients will need frequent molecular monitoring [128]. The ESMO guidelines recommend monthly monitoring in the first six months after TKI discontinuation and three months later on [122]. The NCCN guidelines recommend monthly monitoring in the first year, then every six weeks in the second year, and every 12 weeks after that [123]. These recommendations are stringent, this is why Ross and Hughes proposed monthly monitoring in the first six months, and thereafter every second month [129]. For this purpose, a sensitive qRT-PCR is needed with rapid results, and it is important to have standardised detection limits of qRT-PCR reporting and high-quality tests [22]. Recently, digital PCR (dPCR) has been used to detect *BCR*–*ABL1* mRNA in clinical trials of TFR, and is a predictor for molecular relapse [40,105]. In the ISAV study, the rates of relapse were higher in the dPCR positive group compared to the dPCR negative group [37]. While in KID study, negative dPCR at screening was a predictive factor for sustainable MMR [38]. Moreover, in a study involving 142 CML patients who discontinued TKI, dPCR improved the detection of stable DMR, resulting in better selection of candidates for TFR [130]. Furthermore, Bernardi et al. used dPCR to monitor DMR and assess its value in predicting sustained TFR. As the cut-off value for *BCR*–*ABL1* copies/mL was 0.468 by dPCR patients, TFR sustainability was higher in patients with dPCR of less than 0.468, compared to patients with dPCR greater than 0.468. These results suggest that using dPCR to detect very low levels of *BCR*–*ABL1* is beneficial in selecting suitable candidates for a TFR trial [130].

Claudiani et al. [131] have developed a predictive scoring tool for patients attempting TFR. This includes factors such as the probability of TFR in MR3 (pTFR3) and TFR in MR4 (pTFR4), duration of MR4, previous TKI resistance, age at diagnosis, and transcript type. This could become the new “Sokal score” for modern CML treatment, although further validation is required [131].

## 7. Conclusions

In conclusion, the currently available data on TFR demonstrate that 40–60% of patients who discontinue TKI can maintain a successful TFR. Most relapses occur within the first six months and all patients were sensitive to TKI and gained a molecular response upon treatment re-initiation. The long duration of TKI therapy and maintaining DMR for >2 years were associated with better TFR rates. Furthermore, a high Sokal score, young age, *BCR*–*ABL1* transcript types and level, and TKI resistance indicated the failure of TFR. The predictive score for successful TFR reported by Claudiani et al. may offer reassurance to clinicians when considering discontinuing treatment [131].

In early stopping TKI studies, disease progression was a concern but very few cases of progression have been reported [132,133]. However, it is important to note that the ISAV study reported that those patients who remain in TFR have at least one positive PCR test and using dPCR they estimate that the leukaemic clone may increase by one log over three years; why we do not see continuous growth is unknown and an intriguing scientific question for future studies.

For TFR to become a standardised management goal for CML patients, cure rates need to be high. This can be achieved by optimising the treatment through new therapeutic approaches, and a high quality and sensitive PCR testing should be available with proper and effective monitoring. Clinical trials combining IFN-a and TKI, such as dasatinib plus Peg-IFN alpha-2b and Peg-IFN alpha-2a combined with nilotinib, reported significant DMR rates and patients maintaining better molecular response, although the data on TFR rates are not reported yet. However, it is predicted that this combination may show promising improvements on the outcomes of TFR. Furthermore, increased prospective randomised trials in this field will enable clinicians to address the mechanism behind the molecular biology of TFR. In summary, treatment-free remission is a real and an achievable option for a significant proportion of CML patients, which is arguably a significant milestone in the timeline of CML developments.

## Figures and Tables

**Table 1:** Characteristics of trials discontinuing TKI.

Author	N	Minimum TKI Duration (y)	Type of TKI	Minimum DMR Duration (y)	Median Follow Up (m)	The Median Time to MR (m)	TFR Rate	Treatment Reinitiation
STIM [35,36]	100	3	IM	UMRD ≥ 2	77	2.5	43% at 24 months and 38% at 60 months.	Loss of UMRD
TWISTER [37]	40	3	IM	UMRD ≥ 2	43	3	47.1% at 24 months.	Loss of UMRD
ASTIM [38]	80	3	IM	MR^4^ ≥ 2	31	4	64% at 24 months and 61% at 36 months.	Loss of MMR, UMRD
HOVON [31]	15	2	IM	MR^4.5^ > 2	36	3	33% at 24 months.	Loss of MR^4.5^
ISAV [39]	108	2	IM	UMRD ≥ 1	21.6	NR	48% at 36 months.	Loss of MMR
KID [40]	90	2	IM	MR^4.5^ > 2	26.6	3.3	62.2% at 12 months and 58.5% at 24 months.	Loss of MMR
EURO-SKI [34]	755	3	IM & NL & DA	MR^4^ ≥ 1	27	6	61% at 6 months and 50% at 24 months.	Loss of MRR
DADI (2015) [41]	63	1	DA	DMR ≥ 2	20	3	49% at six months and 48% at 12 months.	Loss of MR^4^
DADI (2020) [42]	58	3	DA	DMR ≥ 2	23.3	2	55% at 6 months.	Loss of MR^4^
ENESTfreedom [43]	190	3	NL	MR^4.5^ ≥ 2	12	NR	51.6 at 48 weeks.	Loss of MMR
Ho-Young Yhim [44]	14	2	IM	DMR ≥ 2	23	NR	28.6% at 12 months.	Loss of MMR
GIMEMA [45]	293	7	IM, NL, DA, BO	DMR ≥ 3	34	3–4	69% at 12 months and 62% at 34 months.	Loss of MMR
STIM2 [46]	124	3	IM	DMR ≥ 2	12	6	61.2% at 12 months.	Loss of MMR
Kieo STIM [47]	40	2	IM	DMR ≥ 2	15.5	NR	55.4% at 12 months.	Loss of MMR
Pagnano KB [48]	48	3	IM	DMR ≥ 2	NR	NR	61% at 20 months.	Loss of MMR
TRAD [49]	67	3	IM	MR^4.5^ ≥ 2	NR	NR	64.7% at 6 months.	Loss of MMR
STIM231 [50]	68	3	IM	MR^4^ ≥ 2	NR	NR	67.6% at 12 months and 65% at 36 months.	Loss of MMR
STOP2G [51]	60	3	NL, DA	MR^4.5^ ≥ 2	47	4	63.3% at 12 months and 53.7% at 48 months.	Loss of MMR
D-STOP [52]	54	2	DA	DMR ≥ 2	16	NR	62.9% at 1 year.	Loss of MMR
DASFREE [53]	84	2	DA	MR^4.5^ ≥ 1	NR	5	48% at 12 months and 46% at 24 months.	Loss of MMR
STATS [54]	78	2	NL	MR^4.5^ ≥ 2	35.4	6	67.9% at 12 months and 62.8% at 24 months.	Loss of MR^4.5^
DOMEST [55]	99	2	IM	MR^4^ ≥ 2	12	NR	70% at 6 months, 68% at 12 months, and 64% at 24 months.	Loss of MR^4^

Abbreviations: IM = Imatinib, DA = Dasatinib, NL = Nilotinib, B = Bosutinib, MR = molecular response, DMR = deep molecular response, TKI = tyrosine kinase inhibitor, MMR = major molecular response, TFR = treatment-free remission, UMRD = undetected minimum residual disease, and NR = not reported.

**Table 2:** Guidelines for TKI discontinuation in clinical practice.

Criteria	FCMLSG [125]	NCCN	ESMO [122]
Age	>18	>18	>18
Phase	CP only	CP only	CP only
Sokal score	not defined	Not defined	Not a high score
*BCR*–*ABL1* transcript	e13a2, e14a2, or e13a2 + e14a2	Quantifiable typical transcript	e13a2, e14a2
TKI duration (years)	>5	>3	>5
DMR type	MR^4.5^	MR^4^	MR^4.5^
DMR duration (years)	>2	>2	>2
Re-treatment	Loss of MMR	Loss of MMR or < MR^4^	Not defined
Prior history	No HSCT, no progression, no resistance, suboptimal response	No prior history of progression or treatment resistance	Optimal response

Abbreviations: CP = chronic phase, TKI = tyrosine kinase inhibitor, DMR = deep molecular response, MMR = major molecular response, MR = molecular response HSCT = haematopoietic stem cell transplant, FCMLSG= French chronic myeloid leukaemia study group, ESMO = European society for medical oncology, and NCCN = national comprehensive cancer network.
